# Survival Rate in Emergency Thoracotomy for Penetrating Trauma: A Retrospective Cross-Sectional Study

**DOI:** 10.7759/cureus.78277

**Published:** 2025-01-31

**Authors:** Abdul Baseer, Muhammad Hammad Khan, Nosheen Noor, Yasir Badshah

**Affiliations:** 1 Department of Thoracic Surgery, Medical Teaching Institute, Lady Reading Hospital, Peshawar, PAK; 2 Department of Radiology, Medical Teaching Institute, Lady Reading Hospital, Peshawar, PAK

**Keywords:** emergency thoracotomy, gunshot wounds, mortality, penetrating trauma survival rate, postoperative outcomes, stab wounds, thoracic injury, trauma

## Abstract

Background

Emergency thoracotomy (ET) is a critical intervention for traumatic thoracic injuries, often required in patients with penetrating trauma. The survival outcomes and the factors that influence survival in these patients remain an area of interest for trauma care providers.

Objective

The objective of the study is to assess the survival rate and factors influencing the survival of patients undergoing emergency thoracotomy due to penetrating thoracic trauma.

Methods

This retrospective cross-sectional study was conducted in the thoracic surgery, accident, and emergency department of Lady Reading Hospital, Peshawar, Pakistan. A total of 85 patients who underwent emergency thoracotomy following penetrating trauma between July 2019 and June 2024 were included. Data were extracted from the health information management system (HIMS) and analyzed using SPSS. The study reviewed demographic details, severity of injury, operative details, and postoperative outcomes. Statistical analyses included univariate analysis using chi-square and Fisher’s exact tests for categorical data and independent t-tests for continuous variables.

Results

The study included 85 patients with a mean age of 25.72±7.84 years. The majority of patients were male (81/85, 95.29%), while females accounted for a smaller proportion (4/85, 4.71%), and gunshot trauma accounted for the most frequent cause of penetrating injury (50/85, 58.8%). The time to thoracotomy was within 60 minutes of admission, with a mean time of 48±16.5 minutes. A total of 61/85 (71.5%) patients had isolated thoracic injuries, and 24/85 (28.2%) had associated injuries, with 24/85 (28.2%) patients requiring concomitant laparotomy. The overall mortality rate was 9/85 (10.5%), with 7/9 (77.7%) of deaths occurring intraoperatively. Mortality was significantly higher in patients with combined thoracotomy and laparotomy procedures. Prolonged hospital stays and higher transfusion requirements were observed in gunshot wound victims (50/85, 58.8%).

Conclusions

Emergency thoracotomy for penetrating thoracic trauma has a significant mortality rate in patients with combined thoracic and abdominal injuries. Factors such as the mechanism of injury, associated injuries, and the need for additional procedures (e.g., laparotomy) influence survival outcomes. Early intervention and proper management of associated injuries are crucial for improving survival rates in these patients.

## Introduction

Thoracic injuries are a significant contributor to trauma-related morbidity and mortality worldwide [1]. In Pakistan, thoracic trauma is a common presentation in tertiary care settings, often resulting from both blunt and penetrating injuries [2]. Compared to blunt thoracic trauma, penetrating injuries are typically associated with higher severity scores, greater complexity, and increased fatality rates. In urban centers, penetrating thoracic injuries (PTIs) are primarily caused by stab wounds or firearm injuries, while blunt trauma is often the result of road traffic accidents and falls. These injuries frequently necessitate prompt surgical intervention, with thoracotomy being a life-saving procedure in select cases [3].

Despite advancements in trauma care, the management of patients requiring emergency thoracotomy remains challenging [4]. The injury severity score (ISS) was developed by Baker et al. in 1974 [5]. It is an anatomic scoring system based on the abbreviated injury scale (AIS). The latter was developed by the Committee on Medical Aspects of Automotive Safety of the American Medical Association in 1971. The AIS has undergone periodic revisions. The ISS is calculated by summing the squares of the three highest AIS scores from different body regions to provide an overall assessment of a patient’s injuries. Notably, a score of six in any region automatically results in a maximum ISS of 75.

Studies have shown the ISS to predict mortality and morbidity accurately in trauma patients. However, the ISS is criticized for two main reasons. The first is that it does not weigh the injury peculiarity when assigning codes. For example, a unilateral flail chest with three or more fractured ribs and a type C pelvic fracture has an AIS score of four each, even though the flail chest is a potentially more severe injury than the fracture. Hence, identical ISS scores in different patients might represent different prognoses. The second is that it underestimates the injury severity in a patient with multiple injuries in one region. For example, a patient with bilateral femoral fractures, skin abrasion, and lip laceration will have scores for one of the fractures in the extremity region, skin abrasion in the external, and lip laceration in the face region. However, femoral fractures are more severe injuries than the latter injuries. Osler et al., in 1997, proposed the new injury severity score (NISS) to address the second deficiency of the ISS [6]. The NISS sums the squares of the three injuries with the highest AIS scores, regardless of the region of the body affected. However, there is limited research focusing on survival rates and the factors influencing outcomes following emergency thoracotomy in Pakistan, particularly in resource-constrained settings [6,7].

The initial assessment of trauma patients prioritizes the evaluation of airway, breathing, and circulation, commonly referred to as the ABCs of trauma care. Imaging plays a crucial role in diagnosing and managing penetrating chest trauma. Chest radiography is often the first imaging modality utilized due to its accessibility, speed, and ability to detect life-threatening conditions such as tension pneumothorax, hemothorax, or mediastinal injuries [8,9].

A portable chest radiograph performed at the bedside provides rapid visualization of injuries and aids in guiding immediate management decisions. However, while these radiographs offer essential preliminary information, their limitations should be acknowledged. Factors such as foreign bodies, suboptimal patient positioning, or inadequate inspiration can compromise image quality, particularly in trauma settings [10]. The rationale of this study is to analyze the survival rates of patients undergoing emergency thoracotomy for penetrating thoracic trauma over a defined period. By examining data from this tertiary care hospital, the study aims to provide insights into the factors influencing survival outcomes and contribute to improving trauma care in Pakistan.

## Materials and methods

This retrospective cross-sectional study was conducted in the thoracic surgery, accident, and emergency department of Lady Reading Hospital, Peshawar, a major referral center serving trauma patients from Khyber Pakhtunkhwa, Pakistan. The hospital is the largest public tertiary care facility in the region, with more than 1500 beds, and receives most trauma cases from the area for definitive diagnosis and treatment. The study reviewed the medical records of 85 patients admitted with thoracic trauma between July 2019 and June 2024.

The sample size of 85 patients was calculated using the OpenEpi calculator, assuming a population proportion of 6% for patients requiring thoracotomy following thoracic trauma, based on prior literature, with a confidence level of 95% and a margin of error of 5%. A simple, convenient sampling technique was employed to include all eligible patients presenting during the study period.

Patients who underwent thoracotomy within 24 hours of sustaining a thoracic injury were included in the study. Patients who underwent thoracotomy for resuscitative or non-emergent reasons were excluded, as were those with isolated abdominal injuries resulting from penetrating thoracic trauma or trans mediastinal injuries causing cardiovascular damage that necessitated emergency sternotomy or thoracotomy.

Ethical approval for the study was obtained from the institutional review board of Lady Reading Hospital (Reference Number 777/LHR/MTI, dated 07/06/2023). Patient confidentiality was strictly maintained by anonymizing data and restricting access to authorized personnel.

Data were retrieved from the hospital’s health information management system (HIMS). Demographic variables included age, gender, and mechanism of injury (stab, firearm, or blunt trauma). In stable patients, chest radiographs and computed tomography (CT) thorax were performed for radiological evaluation. Where concomitant abdominal injury was suspected, CT abdomen was also performed according to hospital emergency protocol. A consultant radiologist reviewed the CT scans of the chest and abdomen. The documented operative details included the indications for thoracotomy, intraoperative findings, and performed procedures. Postoperative outcomes were assessed, noting the length of hospital stay (<7 days or ≥7 days), transfusion requirements, reoperations, and mortality rates.

Patients were managed according to advanced trauma life support (ATLS) protocols. Indications for thoracotomy included an initial chest tube output exceeding 1000 mL, a continuous output of 250 mL/hour for three consecutive hours, large hemothorax accompanied by shock (systolic blood pressure <90 mm Hg), signs of pericardial tamponade, radiological evidence of hemothorax with opacification of more than 50% of lung volume, massive air leak, or hemodynamic deterioration. In all cases, single-lumen endotracheal intubation was performed to achieve rapid airway access.

Data were analyzed using SPSS version 26 (SPSS Inc., Chicago, IL, USA). Continuous variables were expressed as mean±standard deviation (SD), while categorical variables were presented as frequencies and percentages. Univariate analysis for categorical data was performed using the chi-square test or Fisher’s exact test as necessary. Continuous variables were analyzed using an independent paired t-test and Levene’s test for equality of variances. Statistical significance was set at a p-value <0.05.

## Results

Patients with gunshot injuries (n=50) were slightly older (26.62±9.10 years) compared to those with stabbing injuries (n=35; 25.35±9.40 years), but the difference was not statistically significant (p=0.41). The length of hospital stay was significantly longer in gunshot injury patients (12.45±8.60 days) than in stabbing injury patients (9.05±6.50 days, p=0.004). Blood transfusion requirements were higher in the gunshot group, with a mean of 4.55±3.25 units compared to 3.40±1.80 units in the stabbing group (p=0.011). Initial chest tube output was higher in the gunshot group (1060.30±460.80 ml) than in the stabbing group (950.20±430.75 ml), but this difference was not statistically significant (p=0.20). Ongoing chest tube output was slightly higher in stabbing injuries (825.60±356.05 ml) compared to gunshot injuries (722.80±342.15 ml), but the difference was also not statistically significant (p=0.32). No significant difference was found in systolic blood pressure (SBP), with gunshot patients having a mean SBP of 82.50±30.85 mmHg and stabbing patients 78.25±31.65 mmHg (p=0.48). Gender distribution was similar in both groups, with the majority being male (48:2 in gunshot injuries vs. 33:2 in stabbing injuries, p=0.89). Associated injuries were significantly more common in gunshot injuries (22/50, 44.0%) compared to stabbing injuries (13/35, 37.1%; p=0.021) (Table 1).

**Table 1:** Demographic, physiological, and outcome data based on mechanism of injury. SBP:Systolic blood pressure ; continuous variables were analyzed using an independent paired t-test, categorical variables were analyzed using a chi-square test. Results are considered significant at p≤0.05.

Parameters	Gunshot (n=50)	Stabbing (n=35)	p-value
Age (years)	26.62±9.1	25.35±9.4	0.41
Length of hospital stay (days)	12.45±8.6	9.05±6.5	0.004
Blood transfusion requirement (units)	4.55±3.25	3.4±1.8	0.011
Initial chest tube output (ml)	1060.3±460.8	950.2±430.75	0.20
Ongoing chest tube output (ml)	722.8±342.15	825.60±356.05	0.32
SBP (mmHg)	82.5±30.85	78.25±31.65	0.48
Gender ratio (M:F)	48:2	33:2	0.89
Associated injuries (n)	22/50	13/35	0.021

Lung injuries were significantly more frequent in gunshot injuries (42/50, 84.0%) compared to stabbing injuries (20/35, 57.1%; p=0.002). Intercostal artery injuries were more prevalent in stabbing injuries (4/35, 11.4%) compared to gunshot injuries (3/50, 6.0%; p=0.03). Similarly, internal mammary artery injuries were slightly more common in stabbing injuries (2/35, 5.7%) than in gunshot injuries (2/50, 4.0%; p=0.04). Cardiac injuries were observed in 5/35 (14.3%) stabbing cases and 5/50 (10.0%) gunshot cases (p=0.008). Diaphragmatic injuries were present in 4/35 (11.4%) stabbing cases versus 3/50 (6.0%) gunshot cases (p=0.045). Chest wall injuries were significantly more common in gunshot injuries (8/50, 16.0%) compared to stabbing injuries (2/35, 5.7%; p=0.025). Tracheobronchial and vascular injuries were rare in both groups, with no significant differences observed (p>0.05) (Table 2).

**Table 2:** Intraoperative findings based on mechanism of injury. Chi-square test was performed. Results are considered significant at p≤0.05.

Findings	Stabbing Injury (n=35), n (%)	Gunshot Injury (n=50), n (%)	p-value	Chi-square (degrees of freedom=(df)	Effect size (Cohen’s d)
Lung	20 (57.1%)	42 (84.0%)	0.002	9.935 (1)	0.60
Intercostal artery	4 (11.4%)	3 (6.0%)	0.03	4.333 (1)	0.33
Internal mammary artery	2 (5.7%)	2 (4.0%)	0.04	1.568 (1)	0.22
Cardiac	5 (14.3%)	5 (10.0%)	0.008	7.258 (1)	0.45
Tracheobronchial	0 (0%)	2 (4.0%)	>0.05	1.944 (1)	0.12
Diaphragmatic	4 (11.4%)	3 (6.0%)	0.045	3.943 (1)	0.28
Vascular	1 (2.9%)	2 (4.0%)	>0.05	1.436 (1)	0.19
Chest wall	2 (5.7%)	8 (16.0%)	0.025	5.121 (1)	0.34

Lung injury was more common in gunshot injuries (41/50; 82.0%) compared to stabbing injuries (11/35; 31.4%, p=0.03). Other causes of morbidity, including acute respiratory distress syndrome (ARDS), empyema, intra-abdominal abscess, wound infections, and multiple organ failure, were noted; however, no statistically significant differences were observed between the two groups (Table 3).

**Table 3:** Morbidity in patients with penetrating chest trauma. Chi-square test was performed. Results are considered significant at p≤0.05.

Causes of morbidity	Stabbing (n=35), n (%)	Gunshot (n=50), n (%)	Total (n=85), n (%)	p-value	Chi-square (degrees of freedom = (df)	Effect size (Cohen’s d)
Atelectasis	1 (2.9%)	7 (14.0%)	8 (9.4%)	0.02	5.356 (1)	0.47
Acute respiratory distress syndrome (ARDS)	1 (2.9%)	3 (6.0%)	4 (4.7%)	0.04	3.255 (1)	0.29
Pneumonia	1 (2.9%)	5 (10.0%)	6 (7.1%)	0.03	4.774 (1)	0.35
Empyema	1 (2.9%)	1 (2.0%)	2 (2.4%)	0.56	0.337 (1)	0.08
Intra-abdominal abscess	1 (2.9%)	1 (2.0%)	2 (2.4%)	0.56	0.337 (1)	0.08
Intra-parenchymal hematoma	0 (0%)	2 (4.0%)	2 (2.4%)	0.16	2.600 (1)	0.19
Disseminated intravascular coagulation (DIC)	1 (2.9%)	1 (2.0%)	2 (2.4%)	0.56	0.337 (1)	0.08
Deep venous thrombosis (DVT)	1 (2.9%)	1 (2.0%)	2 (2.4%)	0.56	0.337 (1)	0.08
Wound infection	1 (2.9%)	3 (6.0%)	4 (4.7%)	0.02	4.333 (1)	0.33
Anoxic brain injury	1 (2.9%)	2 (4.0%)	3 (3.5%)	0.61	0.497 (1)	0.07
Multiple organ failure	2 (5.7%)	3 (6.0%)	5 (5.9%)	0.88	0.037 (1)	0.04

Mortality was significantly associated with abdominal injuries and intercostal artery injuries. Patients with abdominal injuries had a higher mortality rate (9/40; 22.5%) than those without them (6/45; 13.3%, p=0.02). Notably, there was an absence of mortality in patients with intercostal artery injuries (0/15; 0%) compared to those without intercostal artery injuries (15/70; 21.4%, p=0.04) (Table 4).

**Table 4:** Factors associated with mortality (discrete variables) Chi-square test was performed. Results are considered significant at p≤0.05.

Factors	Mortality, n (%)	p-value	Chi-square (df)	Effect size (Cohen’s d)
Gender		0.68	1.262 (1)	0.14
Male	13/68 (19.1%)			
Female	2/17 (11.8%)			
Mechanism of Injury		0.31	1.019 (1)	0.14
Gunshot	11/50 (22.0%)			
Stabbing	4/35 (11.4%)			
Abdominal Injury		0.02	4.994 (1)	0.36
Present	9/40 (22.5%)			
Absent	6/45 (13.3%)			
Lung Injury		0.07	3.358 (1)	0.27
Present	7/65 (10.8%)			
Absent	8/20 (40.0%)			
Cardiac Injury		0.12	2.467 (1)	0.23
Present	6/25 (24.0%)			
Absent	9/60 (15.0%)			
Intercostal artery injury		0.04	4.093 (1)	0.30
Present	0/15 (0%)			
Absent	15/70 (21.4%)			

Ongoing chest tube output was markedly higher in non-survivors (1502 ml) than in survivors (759.82±319.14 ml, p=0.001), reflecting the impact of uncontrolled bleeding. Moreover, patients who died required significantly more blood transfusions (7.11±4.76 units) compared to those who survived (3.42±1.85 units, p=0.004), highlighting the severity of hemorrhage in influencing mortality. Hemodynamic instability, as reflected by a significantly lower systolic blood pressure (SBP) (43.15±36.45 mmHg in non-survivors vs. 83.67±27.92 mmHg in survivors, p=0.001), further emphasized its role in adverse outcomes (Table 5).

**Table 5:** Factors associated with mortality (continuous variables). SBP: systolic blood pressure, continuous variables were analyzed using an independent paired t-test. Results are considered significant at p≤0.05.

Factors	Mortality (Mean±SD)	Survival (Mean±SD)	p-value
Age (years)	23.89±6.95	26.12±9.47	0.364
Initial output (ml)	1078.42±320.88	997.63±453.25	0.497
Ongoing output (ml)	1502±0.0	759.82±319.14	0.001
Transfused blood (units)	7.11±4.76	3.42±1.85	0.004
SBP (mmHg)	43.15±36.45	83.67±27.92	0.001
Time to operating room (hours)	1.98±1.69	3.25±3.52	0.122

## Discussion

Penetrating thoracic injuries, though not as common as blunt trauma, often present a significant challenge in emergency trauma care. Approximately 10-15% of patients with thoracic trauma require surgical intervention, with the majority of these injuries being penetrating in nature [11,12]. Most penetrating thoracic injuries can be managed through minimally invasive methods, such as chest tube insertion, but some patients inevitably require urgent, operative interventions due to the severity of the injury or associated complications [13,14]. Although chest tube thoracostomy is a life-saving procedure, it is still considered an invasive intervention that carries potential life-threatening risks and complications. Clinicians performing this procedure should be well-trained to identify and address these complications effectively. In this study, 14.06% (12/85) of patients with penetrating chest injuries required an immediate thoracotomy, a rate that aligns with other reports in the literature. An immediate thoracotomy can be performed in the operating theater, herein referred to as an emergency thoracotomy (ET), or at the emergency department (ED), herein referred to as an emergency department thoracotomy (EDT) [15].

Penetrating trauma accounts for the majority of cases that necessitate thoracotomy in civilian trauma settings, a trend that has been shaped by historical military experiences during World War II, the Korean War, and the Vietnam War, which provided early insights into the indications for thoracotomy following trauma [16]. In the civilian context, these indications include the need to control persistent chest output, continuous hemorrhage, tamponade, massive hemothorax, and massive air leaks, which are associated with severe vascular damage [17].

Our findings corroborate those of previous studies, which suggest that a significant amount of blood loss through chest tube drainage is not always indicative of the severity of chest injuries [18]. For instance, although initial blood loss through chest tubes may exceed 1000 ml or continue at 250 ml/hour for three hours, this alone is not always a reliable predictor of severe thoracic injury. While Karmy-Jones et al. observed that continuous chest output is linked to increased mortality. Their study included both blunt and penetrating injuries [19]. In our series, a similar correlation was observed between ongoing chest output and increased risk of death. Moreover, some studies have highlighted the advantages of performing thoracotomies in the operating room due to better lighting, advanced surgical equipment, and the presence of skilled surgical teams [20]. Although certain trauma centers have seen better outcomes with thoracotomies performed in an operating room setting, we found no significant differences in survival rates between the locations of the thoracotomy, whether in the emergency department or the operating room, specifically in cases of stab wounds. However, the complexity of gunshot wounds, which often involve multiple organ systems, may necessitate a more intricate approach and require thoracotomies performed in a controlled operating room environment [21].

The type of incision made during the procedure plays a significant role in the approach to penetrating chest injuries. In our study, anterolateral thoracotomies (the incision is made in the fourth or fifth intercostal space. The incision for anterolateral thoracotomy begins slightly lateral to the sternum and extends laterally toward the midaxillary line, following the natural contour of the ribs to optimize exposure and access. This approach was most commonly performed for stab wounds, accounting for 57% (20/35) of cases. In contrast, posterolateral thoracotomy, where the incision is made in the fifth or sixth intercostal space depending on the injury location and required access, starts posteriorly just below the scapula and curves along the rib to the midaxillary line. This technique was more frequently used for gunshot wounds, comprising 80% (40/50) of cases. According to advanced trauma life support (ATLS) guidelines, thoracic surgical intervention is recommended when there is blood loss exceeding 1,500 mL at first or more than 200 mL/hour during the initial 2-4 hours following chest tube placement, endobronchial blood loss or tracheobronchial injury, or injuries to the heart or great vessels.

Additionally, the eastern association for the surgery of trauma advises thoracotomy in cases of penetrating trauma with or without vital signs but discourages it in cases of blunt trauma without vital signs. These recommendations emphasize that outcomes following emergency thoracotomy heavily rely on appropriate patient selection. Although some authors recommend an extension of the incision for better exposure, our study did not require an incision extension. For cardiac injuries, which are more likely to present with signs of tamponade or direct damage, an anterior thoracotomy or median sternotomy provides the best access [22]. If necessary, sternotomy allows for easier access to the right hilum and facilitates cardiopulmonary bypass if cardiac repair is required [23].

Regarding lung injuries, approximately 70.5% (60/85) of patients in our cohort required pulmonary parenchymal surgery, a rate higher than 40%. The surgical approach for these injuries often involves tractotomy or simple suture techniques, which were employed in 68% (41/60) and 23% (14/60) of cases, respectively. Wedge resection, though less common, was performed in 9% (5/60) of patients. Interestingly, lung injuries did not significantly affect the mortality rate in our study (p=0.09), which suggests that the severity of parenchymal injury may not directly correlate with patient outcomes. This contrasts with previous studies that found higher mortality rates associated with lobectomy or pneumonectomy following traumatic lung damage [24]. Our approach, focusing on less invasive resection techniques, is consistent with the evolving trend in thoracic trauma surgery that prioritizes lung preservation and minimizes the need for major resection procedures.

The overall mortality rate in our study was 10.8% (9/85), with a notable increase in mortality among patients requiring combined thoracotomy and laparotomy procedures, who had a death rate of 25% (6/24). On average, patients in our study required 2.8 units of blood transfusions, with a range of 1 to 6 units, depending on the severity of their injuries. However, our death rate was lower than that reported in studies with a higher proportion of gunshot wounds. Moreover, mortality rates were significantly influenced by clinical factors such as systolic blood pressure at presentation, the need for blood transfusions, and injury severity, which were correlated with poorer outcomes [25-28].

In terms of management, surgeons must be vigilant in recognizing signs of life-threatening injuries, including massive hemothorax, pericardial tamponade, and major vascular damage, all of which necessitate prompt surgical intervention. Factors such as initial blood loss, continued chest output, and associated injuries (particularly abdominal injuries) are important predictors of mortality [29]. Our study found that the presence of concurrent abdominal injuries was associated with a significant increase in mortality, rising from 5.3% (3/61) to 25% (6/24). This underscores the complexity of managing penetrating chest trauma, where the combination of thoracic and abdominal injuries often results in worse outcomes. Surgeons must, therefore, maintain flexibility in their surgical approach and be prepared for the unexpected challenges posed by penetrating trauma, which often requires rapid decision-making and the ability to adapt to the evolving clinical situation.

Limitations

This study has several limitations. Firstly, its retrospective design limits the ability to establish causality, relying on pre-existing data that may contain biases or missing information. The single-center nature of the study restricts the generalizability of the findings to other settings. Data completeness could also be an issue due to potential inaccuracies or missing entries in the HIMS. Furthermore, unmeasured confounders, such as comorbidities, pre-hospital care, and the surgical team's experience, were not accounted for. The lack of long-term follow-up restricts insights into patient recovery and long-term outcomes, and the study did not consider variations in surgical techniques that may influence survival rates.

## Conclusions

While the management of penetrating thoracic trauma continues to evolve, the importance of prompt recognition of critical injuries and timely surgical intervention cannot be overstated. Our findings emphasize the significance of tailored surgical approaches based on the nature of the injury, whether stab or gunshot, and highlight the complexity of managing combined thoracic and abdominal trauma. No emergency thoracotomy should be performed if no signs of life are present on the initial pre-hospital field assessment. However, emergency thoracotomy remains an indicated procedure in most patients sustaining penetrating trauma, provided there are clinical signs of life. In contrast, blunt traumatic cardiac arrest serves as a relative contraindication to emergency thoracotomy, given its poor prognosis and limited utility in such scenarios. Mortality remains high among patients with multiple organ injuries, especially when surgical expertise and the use of advanced techniques such as video-assisted thoracoscopic surgery are limited in certain trauma settings. 
